# Long Noncoding RNA Expression Signatures of Colon Cancer Based on the ceRNA Network and Their Prognostic Value

**DOI:** 10.1155/2019/7636757

**Published:** 2019-03-11

**Authors:** Yang Cheng, Lanlan Geng, Kunyuan Wang, Jingjing Sun, Wanfu Xu, Sitang Gong, Yun Zhu

**Affiliations:** ^1^Liver Tumor Center, Department of Infectious Diseases and Hepatology Unit, Nanfang Hospital, Southern Medical University, Guangzhou, Guangdong 510515, China; ^2^Digestive Department, Guangzhou Women and Children's Medical Center, Guangzhou Medical University, Guangzhou, Guangdong 510623, China

## Abstract

**Background:**

The specific functional roles of long noncoding RNAs (lncRNAs) as ceRNAs in colon cancer and their potential implications for colon cancer prognosis remain unclear. In the present study, a genome-wide analysis was performed to investigate the potential lncRNA-mediated ceRNA interplay in colon cancer based on the “ceRNA hypothesis.” The prognostic value of the lncRNAs was evaluated.

**Methods:**

A dysregulated lncRNA-associated ceRNA network was constructed based on the miRNA, lncRNA, and mRNA expression profiles in combination with the miRNA regulatory network by using an integrative computational method. Molecular biological techniques, including qPCR and gene knockdown techniques, were used to verify candidate targets in colon cancer. Survival analysis was performed to identify the candidate lncRNAs with prognostic value.

**Results:**

Our network analysis uncovered several novel lncRNAs as functional ceRNAs through crosstalk with miRNAs. The QRT-PCR assays of patient tissues as well as gene knockdown colon cancer cells confirmed the expression of top lncRNAs and their correlation with target genes in the ceRNA network. Functional enrichment analysis predicted that differentially expressed lncRNAs might participate in broad biological functions associated with tumor progression. Moreover, these lncRNAs may be involved in a range of cellular pathways, including the apoptosis, PI3K-AKT, and EGFR signaling pathways. The survival analysis showed that the expression level of several lncRNAs in the network was correlated with the prognosis of patients with colon cancer.

**Conclusions:**

This study uncovered a dysregulated lncRNA-associated ceRNA network in colon cancer. The function of the identified lncRNAs in colon cancer was preliminarily explored, and their potential prognostic value was evaluated. Our study demonstrated that lncRNAs could potentially serve as important regulators in the development and progression of colon cancer. Candidate prognostic lncRNA biomarkers in colon cancer were identified.

## 1. Introduction

Colon cancer is one of the most common cancers in the world. In developing countries, approximately one-quarter of the patients suffering from colon cancer are in an advanced stage at presentation and have lost the opportunity for radical surgery [1, 2]. It is important to search for prognostic biomarkers and new therapies for human colon cancer.

Noncoding RNAs (NcRNAs) have appealed to researchers due to the modulating effect on the biological behaviors of tumor cells [3, 4]. Among these NcRNAs, long noncoding RNAs (lncRNAs) are a main focus of attention. Increasing evidence has revealed that lncRNAs possess significant regulatory effects on carcinogenesis and tumor development [5–8].

The competing endogenous RNA (ceRNA) hypothesis was first proposed by Salmena and colleagues, who suggested that protein-coding RNAs and NcRNAs act as ceRNAs by competing for miRNAs through shared miRNA response elements to mutually regulate their expression [9]. ceRNA has received wide attention as a novel approach to regulating genes. Given the prominent functions of ceRNAs in physiology, their deregulation is a common occurrence in cancer that can promote progression [10–12]. lncRNAs can act as ceRNAs to sponge miRNAs and prevent these miRNAs from binding to mRNAs, thus regulating target genes posttranscriptionally [13]. Systematic analysis focused on lncRNA-associated ceRNA networks has been performed in a variety of cancers [14–19]. However, the complexity and behavior of the lncRNA-associated ceRNA network remain poorly understood in the progression of colon cancer.

Here, we used an integrative computational method to identify lncRNA-mRNA-related crosstalk networks mediated by miRNAs based on the “ceRNA hypothesis” using data from The Cancer Genome Atlas (TCGA). Candidate prognostic lncRNA biomarkers in colon cancer were identified. The expression of candidate lncRNAs and target genes was also confirmed in clinical colon cancer tissues and colon cancer cell lines.

## 2. Materials and Methods

### 2.1. Data Collection

Data from patients with colon cancer were downloaded from TCGA data portal website (available at https://cancergenome.nih.gov/) [20, 21]. Data on 439 tumorous tissues and 42 nontumorous adjacent tissues from 439 colon cancer patients with clinical follow-up information were included. The detailed characteristics of the included patients are shown in Doc S1. The mRNA and lncRNA expression profile data were derived from the TCGA COAD RNA-sequencing dataset. The miRNA expression profile data was derived from the TCGA COAD miRNA-sequencing dataset.

The human miRNA and targeted gene data were collected from miRTarBase (version 6.1) [22]. The data on human miRNA targeting lncRNA were collected from LncBase (version 2) [23].

### 2.2. Identification of Differentially Expressed lncRNAs, mRNAs, and miRNAs

The differentially expressed mRNAs, lncRNAs, and miRNAs in normal colon tissues and colon cancer tissues in the TCGA were analyzed using edgeR software packages. The false discovery rate (FDR) was controlled at the 0.01 threshold (Benjamini and Hochberg algorithm). The fold-change (FC) threshold was set at more than 2.0.

### 2.3. miRNA Target Prediction

miRTarbase is an information resource for experimentally validated miRNA-target interactions that provides the expression profile of a miRNA and its target gene. For the differentially expressed genes in TCGA, high-quality experimentally validated miRNA-target gene interaction relationships from published low-throughput experiments were selected. A total of 1523 pairs of miRNA-gene interactions containing 345 miRNAs and 531 genes were selected.

LncBase provides experimentally supported and in silico predicted interactions of miRNA and lncRNAs. For the differentially expressed lncRNAs, target miRNA-lncRNA in LncBase with a score > 0.7 were selected. A total of 1025 pairs of miRNA-lncRNA interactions containing 492 miRNAs and 132 lncRNAs were selected.

### 2.4. Construction of lncRNA-Associated ceRNA Network

The Pearson correlation coefficient (PCC) for miRNA-mRNA and miRNA-lncRNA was calculated using paired miRNA, mRNA, and lncRNA expression profile data. A candidate pair of lncRNA-miRNA-mRNA was constructed based on the “ceRNA hypothesis” as follows: (i) mRNAs and lncRNAs share the same miRNAs; (ii) mRNAs and lncRNAs suggest a positive correlation when the PCC is higher than 0.3 and *P* value < 0.05; (iii) mRNAs and lncRNAs show a negative regulation with miRNA with PCC < 0 and *P* value < 0.05; and (iv) the miRNAs are aberrantly expressed in colon cancer.

All of the functional pairs were integrated to form a miRNA-mediated, lncRNA-associated ceRNA network. The degree values represent the number of genes with which the lncRNA can interact. The higher the degree is, the more centrally the lncRNA occurs within the network.

### 2.5. Gene Ontology Analysis

The Gene Ontology (GO) analysis was used to determine the potential roles of aberrantly expressed lncRNAs in colon cancer. GO enrichment was performed using BiNGO: a Cytoscape plugin (http://cbl-gorilla.cs.technion.ac.il) [24].

### 2.6. KEGG Pathway Analyses

The Kyoto Encyclopedia of Genes and Genomes (KEGG) pathway analysis (https://www.kegg.jp/) was performed to gain insight into the underlying biology of the ceRNA network as well as the top five lncRNAs with the highest degree in the ceRNA network. A clusterProfiler R package was used for the enrichment analysis [25].

### 2.7. Survival Analysis

A Kaplan-Meier survival analysis was performed for patients with different lncRNA expressions. Statistical significance was assessed using the log-rank test. Patients were assigned to high-/low-expression groups according to the median of the expression level of lncRNAs. A flowchart of the ceRNA network construction and analysis is shown in Figure S1.

### 2.8. Patient Tissue Sample Preparation

Tissue samples of colon cancer were obtained from surgical specimens at Nanfang Hospital. The specimens were snap-frozen in liquid nitrogen after excision. Thirty-five samples of colon cancer tissues and paired adjacent normal colon tissues were used for the lncRNA microarray analysis. The experimental protocols were approved by the Ethics Committee of Nanfang Hospital.

### 2.9. Cell Culture and Transient Transfection

Human colon cancer cell lines HT-29 and HCT116 were obtained from Shanghai Advanced Research Institute, Chinese Academy of Sciences. The cells were cultured using conventional methods. For transient transfection of siRNA, the cells were seeded in 6-well plates and transfected with siRNA using Lipofectamine 2000 (Invitrogen, USA) according to the manufacturer's instructions. The target sequences for siRNAs are shown in Table S1.

### 2.10. Quantitation of the lncRNAs and Target Genes

Total RNA was extracted from the frozen tissues of patients or treated cells using TRIzol reagent (Invitrogen, Carlsbad, CA, USA) following the manufacturer's protocol. Purified total RNA was reverse transcribed using the PrimeScript RT reagent kit (Takara, Japan). Quantitative real-time PCR (qPCR) of the top 5 lncRNAs was performed using the Power SYBR Green PCR Master Mix (lnRcute lncRNA qPCR detection kit) according to the manufacturer's guidelines. The specific lncRNA primers are listed in Table S2. Quantitative real-time RT-PCR of the target genes was performed by specific gene primers using Thermal Cycler Dice Real Time (Takara Bio Inc.) according to the protocol. The specific gene primers are listed in Table S3. The expression levels of mRNA were normalized to the internal control GAPDH and then calculated with the ^Δ^CT method. All data were expressed as the mean ± standard error of the measurement from at least three experiments.

### 2.11. Quantitation of miRNA Expression

Total RNA from cultured cell lines was isolated using the TRIzol reagent (Invitrogen). Reverse transcriptase reactions were performed using Taqman Reverse Transcription Reagents (P/N: N808-0234, Applied Biosystems). Each reaction sample contains total RNA, 50 nM stem-loop RT primer, 1x RT buffer, 0.25 mM dNTPs, 3.33 U/*μ*l MultiScribe reverse transcriptase, and 0.25 U/*μ*l RNase inhibitor. Reactions were performed according to the manufacturer's protocol. Real-time PCR was performed using a standard TaqMan PCR kit protocol. The 10 *μ*l PCR included 0.67 *μ*l RT product, 1x TaqMan Fast Advanced Master Mix (P/N: 4444557, Applied Biosystems), 0.2 *μ*M TaqMan probe, 1.5 *μ*M forward primer, and 0.7 *μ*M reverse primer. The reactions were incubated according to the manufacturer's protocol. The specific miRNA primers are listed in Table S4. All reactions were run in triplicate. The data were normalized to the geometric mean of housekeeping snRNA U6 and calculated as 2^−ΔΔ^CT.

## 3. Results

### 3.1. Identification of Differentially Expressed lncRNAs, miRNAs, and mRNAs

A total of 439 tumorous tissues and 42 nontumorous adjacent tissues were included. A total of 16266 genes, 2758 lncRNAs, and 298 miRNAs were included in our study. In total, 115 miRNAs, 4188 mRNAs, and 663 lncRNAs were identified as differentially expressed between tumor tissues and normal tissues. Of the significantly differentially expressed lncRNAs, 441 were upregulated, and 222 were downregulated. Of the aberrantly expressed mRNAs, 2174 were upregulated, and 2014 were downregulated. In the significantly differentially expressed miRNAs, 71 were upregulated, and 44 were downregulated.

### 3.2. Global Properties of Colon Cancer-Specific ceRNA Network

An experimental interaction network among miRNAs, mRNAs, and lncRNAs was constructed. Positively and negatively correlated mRNA/miRNA pairs are both interesting from a purely statistical framework [26]. However, a high positive correlation between RNAs competing for miRNA binding has been recently experimentally observed and discussed [13]. Thus, we focused on the mRNA/lncRNA pairs that negatively correlated with the shared miRNA.

**Figure 1:**
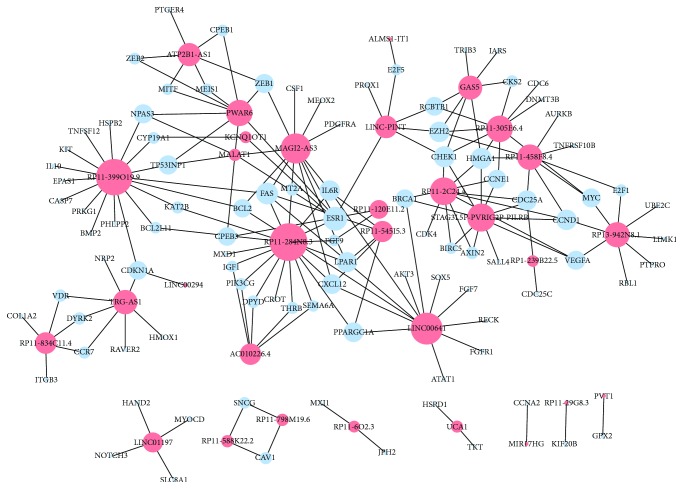
Colon cancer-specific lncRNA-associated ceRNA network. Graphical view of the mRNA-lncRNA network in colon cancer. The size of the nodes represents the power of the interrelation among the nodes. In the network, genes are colored in green, and lncRNAs are colored in red. The more edges a lncRNA has, the more genes that connect to it and the more central a role the lncRNA plays within the network.

**Figure 2:**
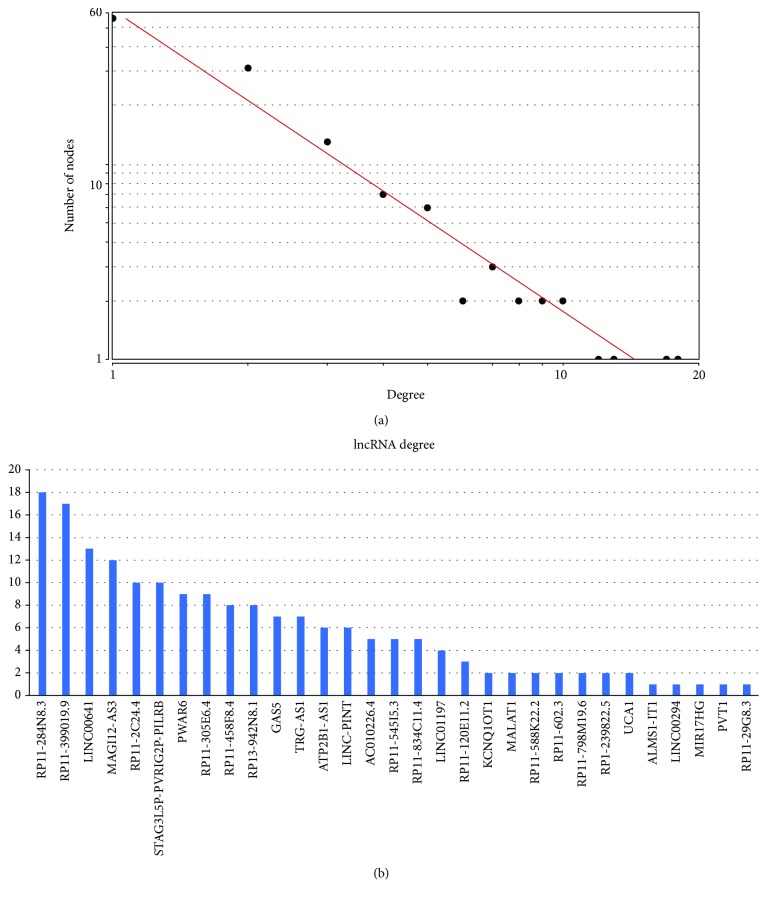
Characteristics of the ceRNA network. (a) Degree distribution of the nodes in the colon cancer-associated ceRNA network. The degree of a node is the number of edges connecting to other nodes. (b) Degree of the lncRNAs in the colon cancer-associated ceRNA network.

A lncRNA-miRNA-mRNA ceRNA network containing 181 ceRNA crosstalk candidates was identified comprising 97 mRNAs, 31 lncRNAs, and 34 miRNAs (Doc S2). The constructed ceRNA network contained 128 nodes and 181 edges (Figure 1). A network analysis of the architecture and features of the colon cancer-specific network was performed (Table S5). The degree of nodes in the ceRNA network was calculated (Figure 2(a)). Most of the nodes had a low degree, and a small portion of nodes had a high degree. The degrees for the 31 lncRNAs are shown in Figure 2(b). The corresponding competing genes of the lncRNAs are listed in Table 1. We found that a variety of tumor progression-related genes were involved, such as apoptosis-related genes (FAS, BCL2, and BCL2L11) and a gene associated with DNA repair (BRCA1).

Notably, lncRNA TRG-AS1 and RP11-399O19.9 in the network were significantly downregulated in the tumor tissues compared with normal colon tissues, and expression of the two lncRNAs was markedly lower in late-stage patients (stages III and IV) than in early-stage patients (stages I and II). Some other lncRNAs (UCA1, RP11-458F8.4, RP1-239B22.5, and ALMS1-IT1) that were upregulated in cancers showed a higher expression in the late stage than in the early stage (Table 2). These results suggest that the expression of these lncRNAs may be involved in the progression of colon cancer.

### 3.3. Validation of the lncRNA and Target Gene Expression Using qPCR

**Figure 3:**
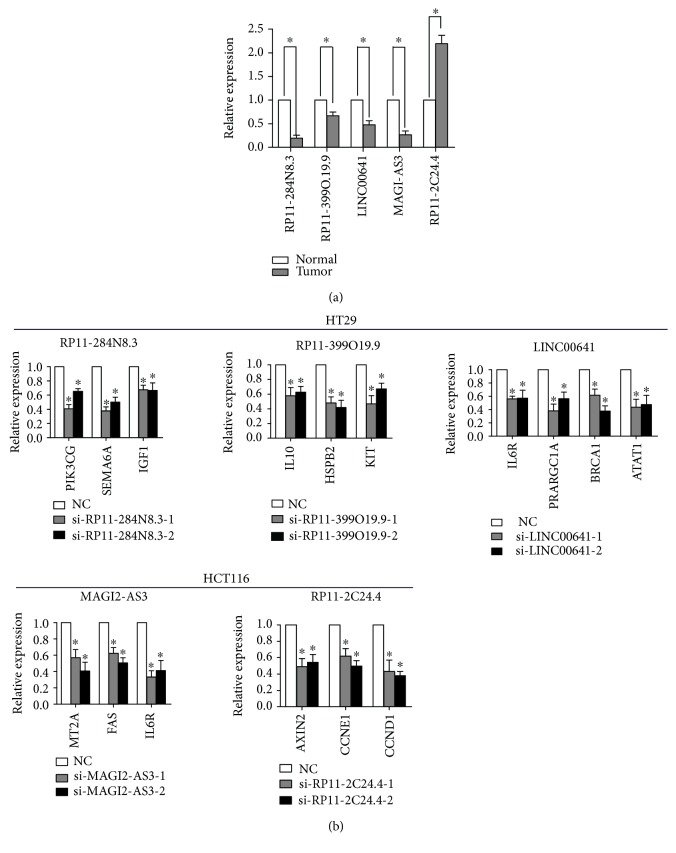
Validation by qPCR of the top five candidate lncRNAs and the correlation between target genes. (a) The relative expression levels of the top 5 lncRNAs with the highest degree in the ceRNA network were detected by qPCR in the tumor tissues and paired adjacent normal colon tissues from 35 patients with colon cancer. The data are presented as the relative expression level in tumor tissues compared with normal control tissues. ^∗^ *P* < 0.05 vs. normal. (b) The colon cancer cell line HT-29 was transfected with siRNA of RP11-284N8.3 and RP11-399O19.9, LIN00641, or the control siRNA. HCT116 was transfected with siRNA of MAGI2-AS3, RP11-2C24.2, or the control siRNA. QPCR was used to detect the expression level of the target genes. NC: negative control. The data are expressed as the mean ± standard error of measurement from at least three experiments. ^∗^ *P* < 0.05 vs. NC.

To verify the reliability of the aberrant lncRNAs found in the TCGA database, a qPCR assay was used to detect the expression levels of the top five lncRNAs with the highest degree in 35 paired tumor tissues and adjacent normal tissues from patients with colon cancer. The results indicate that the RP11-2C24.4 levels increased while the RP11-284N8.3, RP11-399O19.9, LINC00641, and MAGI2-AS3 levels decreased in the tumor tissues (Figure 3(a)). The results were consistent with the expression trends of the data identified in the ceRNA network.

The expression of the involved miRNAs, which were predicted to be “sponged” by the top five lncRNAs in the cell lines, was verified. We showed that all of the miRNAs are expressed in HT-29 and HCT116 cells (Figure S2). Then, we observed whether these lncRNAs and targeted gene expression levels are associated in the colon cancer cell lines HT-29 and HCT116. Small interfering RNAs (siRNA) against the top five lncRNAs were transfected into HT-29 and HCT116 cells. The QRT-PCR analysis revealed that transfection of siRNA reduced the main target genes as revealed by our ceRNA network (Figure 3(b)). For example, our ceRNA network showed that PIK3CG, SEMA6A, and IGF1 are the top three genes that interact with lncRNA RP11-284N8.3 Furthermore, siRNA silencing of RP11-284N8.3 in cancer cells led to decreases in PIK3CG, SEMA6A, and IGF1 mRNA (Figure 3(b)).

### 3.4. Functional Characterization and Pathway Analysis of Colon Cancer-Specific ceRNA Network

**Figure 4:**
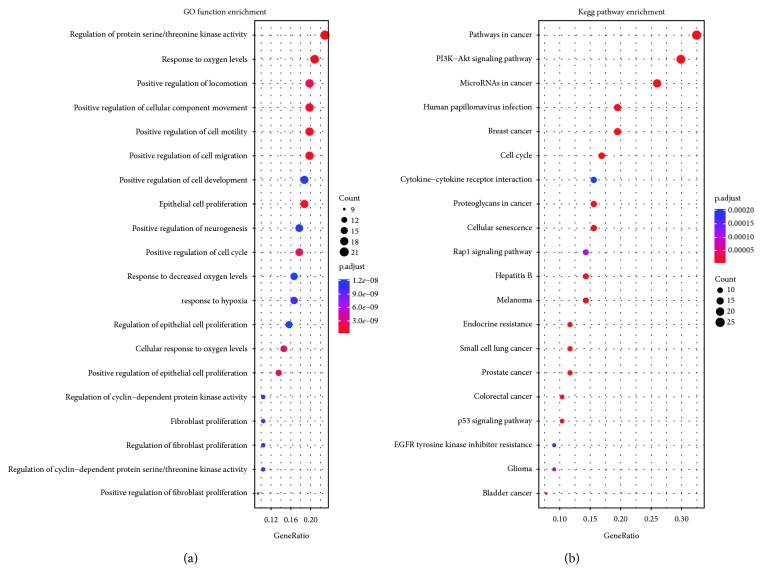
Functional characterization of colon cancer-specific ceRNA network. (a) GO enrichment analysis of the ceRNA network. The top twenty significantly enriched GO terms of biological processes are shown. (b) KEGG pathway analysis of the ceRNA network. The top twenty pathways are shown. The adjusted *P* values for multiple enrichment testing using the Benjamini and Hochberg method are shown. The color represents the significance of enrichment.

To characterize the function of our ceRNA network, a GO enrichment analysis and KEGG pathway analysis were performed (Doc S3 and S4). The top twenty GO functions and KEGG pathways are shown in Figures 4(a) and 4(b). The GO enrichment analysis demonstrated that the ceRNA network is mainly involved in processes such as the regulation of protein serine/threonine kinase activity, response to oxygen levels, the regulation of cell motility, epithelial cell proliferation, and regulation of the cell cycle. The functional pathway analysis revealed that multiple signaling pathways in cancer were enriched in the ceRNA network, including the PI3K-AKT, Rap1, and p53 signaling pathways. The results suggest that the ceRNA network potentially modulated a variety of functions and regulated multiple signaling pathways during colon cancer development.

### 3.5. Mechanism of the Dysregulated lncRNAs in the Colon Cancer-Specific ceRNA Network

**Figure 5:**
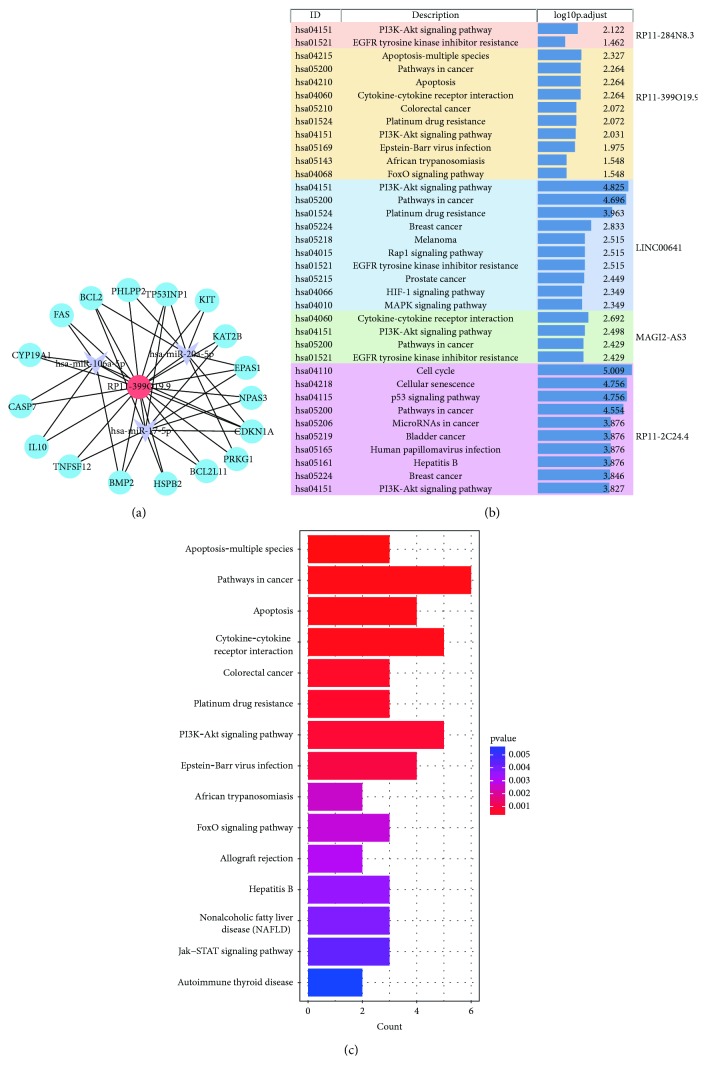
Analysis for lncRNAs in the ceRNA network. (a) Network map for lncRNA RP11-399O19.9-related genes and miRNAs in the ceRNA network. Genes are colored in blue, and miRNAs are colored in purple. (b) KEGG analysis for the top five lncRNAs in the ceRNA network. (c) Significantly enriched KEGG pathway for lncRNA RP11-399O19.9. The top 10 enriched pathways are listed.

To preliminarily explore the potential functions of the dysregulated lncRNAs in colon cancer, a functional enrichment analysis for the target genes of the top 5 lncRNAs with the highest degree was performed based on GO terms. We showed that the mRNA targets for lncRNA RP11-399O19.9 included a variety of tumor-associated genes, such as the apoptosis-related genes FAS, BCL2, and BCL2L11 (Figure 5(a)). The gene function of lncRNA RP11-399O19.9 was significantly enriched in the GO terms mainly involved in cell death and apoptosis, cell cycle, morphogenesis, and response to stimuli (Figure S3). Similar results were found in the GO analysis for lncRNA RP11-284N8.3 (Table 3).

Mechanisms of the dysregulated lncRNAs in colon cancer were further explored using KEGG pathway analysis. The pathway analysis revealed that these lncRNAs participated in several cancer-related signaling pathways including apoptosis, the PI3K-AKT signaling pathway, and the EGFR signaling pathway (Figure 5(b)). Notably, the main enriched signaling pathways regarding lncRNA RP11-399O19.9 contain colon cancer and platinum drug resistance (Figure 5(c)). These results suggest that lncRNAs in the ceRNA network participated in broad biological functions associated with colon cancer.

### 3.6. Progression-Related Network Analysis Reveals Prognostic lncRNA Biomarkers Associated with Colon Cancer

**Figure 6:**
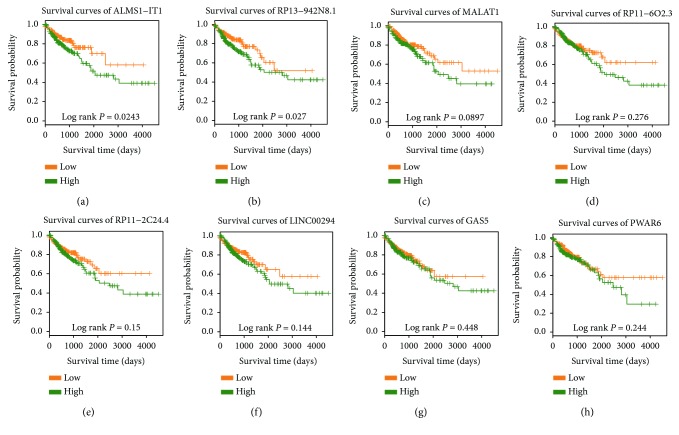
Prognostic value of lncRNAs for assessing the clinical outcome of colon cancer. Kaplan-Meier survival curves for colon cancer patients using the lncRNA signature. Patients were assigned to high-/low-expression groups according to the median of the expression level of lncRNAs. *P* values were calculated using the log-rank test.

We further evaluated the prognostic value of the identified lncRNAs in colon cancer. We found that, compared with lower expression, higher expression of ALMS1-IT1 and RP13-942N8.1 was significantly correlated with poor prognosis of colon cancer (Figures 6(a) and 6(b)). Moreover, low expression of several other lncRNAs including MALAT1, RP11-6O2.3, RP11-2C24.4, LINC00294, GAS5, and PWAR6 correlated with a better prognosis for colon cancer; however, the results are not statistically significant (Figures 6(c)–6(h)). In addition, the expression pattern of the six lncRNAs in the patients is in accordance with the results of the survival analysis, which show higher expression levels in colon cancer tissues than in adjacent normal tissues. These data suggested that these dysregulated lncRNAs might be potential prognostic biomarkers for colon cancer.

## 4. Discussion

The ceRNA hypothesis represents a novel posttranscriptional regulatory dimension of gene regulation [27]. However, interactions between lncRNAs and mRNAs have never been reported in colon cancer. There is also a lack of research investigating changes in the expression of regulatory lncRNAs. To elucidate the important roles of lncRNAs and explore the regulatory networks in colon cancer, a genome-wide analysis of lncRNAs, miRNAs, and mRNAs from the TCGA database was performed. Based on the ceRNA hypothesis, we constructed a lncRNA-associated ceRNA network in colon cancer. Most studies for identifying miRNA sponge interactions use putative miRNA target information and hypergeometric tests to identify candidate miRNA sponges [26, 28–30], whereas our study used experimentally validated miRNA-target interactions. Compared with the methods used in former studies, the method in our study is stricter. Furthermore, molecular biological techniques were performed to verify the expression of candidate lncRNA target genes in colon cancer.

Functional studies show that ceRNA appears to participate in several cancer-related processes such as cell proliferation, cell cycle, and cell invasion. The pathway analysis suggested that the ceRNA network is potentially involved in multiple signaling pathways, such as the PI3K-AKT, Rap1, Ras, cytokine-cytokine receptor interaction, and EGFR tyrosine kinase inhibitor resistance signaling pathways.

Studies have revealed a critical role of lncRNA dysregulation in modulating gene expression as well as tumor development and progression. A recent study reported that lncRNA H19 is a miRNA 200a sponge that inhibits miRNA 200a functions, thereby promoting cell proliferation in colorectal cancer [31]. The report revealed the potential functional significance of the lncRNA-associated ceRNA network in colon cancer. Our analysis revealed that lncRNAs participate in a complex ceRNA network in colon cancer. In total, 31 lncRNAs were found in the network. The QPCR assays of the top five lncRNAs in paired tumor and normal tissues verified the expression trends of the lncRNAs in the ceRNA network. The QRT-PCR analysis also revealed that transfection of siRNA of lncRNAs in the colon cancer cell lines reduced the main target genes revealed by our ceRNA network.

In the constructed ceRNA network, lncRNAs TRG-AS1 and RP11-399O19.9 were significantly downregulated in tumor tissues, and the expression was much lower in late-stage cancer than in early-stage cancer, suggesting that they may be protective factors in colon cancer. Meanwhile, lncRNAs UCA1, RP11-458F8.4, RP1-239B22.5, and ALMS1-IT1, which were markedly upregulated in tumor tissues, have higher expression levels in late-stage cancer. The results suggest that these lncRNAs may play crucial roles in colon cancer occurrence and progression.

From our literature review, we found that several lncRNAs among the ceRNA network, such as lncRNAs UCA1, GAS5, MALAT1, and PVT1, are associated with oncogenesis and the development of colon cancer [32–35]. For example, lncRNA UCA1 influences cell proliferation, apoptosis, and cell cycle distribution, leading to chemoresistance of 5-Fu via competing miRNA-204 functions. lncRNA GAS5 contributes to lymphatic metastasis and was significantly associated with the susceptibility and progression of colon cancer [36]. lncRNA MALAT1 was upregulated in colon cancer tissues and may mediate HMGB1 by sponging miR-129-5p in colon cancer. High expression of lncRNA MALAT1 suggests poor prognosis in colon cancer [35, 37]. lncRNA PVT1 functions as an oncogene in human colon cancer, which could promote the metastasis and proliferation of colon cancer by suppressing miR-30d-5p [38]. In addition, PVT1 overexpression in colon cancer cells significantly promoted cisplatin resistance in vivo [39]. All of these studies support our results and confirmed the role of lncRNAs among the ceRNA network during the development of colon cancer.

However, the functions of most lncRNAs enrolled in the network have not been determined. Computational methods for predicting the function of lncRNA have shown many advantages to functional annotation [40, 41]. An endogenous competing mRNA target was used to predict the functions of lncRNAs. We showed that these mRNA targets for lncRNAs in the ceRNA network included a variety of tumor-associated genes, such as the apoptosis-related genes FAS, BCL2, and BCL2L11 and the DNA repair gene BRCA1. The GO enrichment analysis indicated that these lncRNAs may be involved in a number of biological processes, including cell death and apoptosis, cell cycle, morphogenesis, and response to stimuli. The KEGG pathway analysis suggested that the inferred functions of the top lncRNAs were involved in the apoptosis signaling pathway, PI3K-AKT pathway, EGFR pathway, colon cancer pathway, and platinum drug resistance, which are fundamental processes for colon cancer development.

lncRNAs can be powerful predictors for the survival of cancer patients [42–44]. However, few studies have been performed to predict colon cancer-specific lncRNAs as a biomarker for colon cancer prognosis. This study showed that several lncRNAs in the ceRNA network were correlated with survival prognosis in patients with colon cancer. For example, higher expression of lncRNAs ALMS1-IT1 and RP13-942N8.1 was significantly correlated with poorer survival prognosis in patients. Our discovery indicated that lncRNAs in the constructed ceRNA network might be used to predict the prognosis of colon cancer.

In conclusion, the present study represents a view of colon cancer from a concurrent analysis of lncRNAs, miRNAs, and mRNAs. The constructed colon cancer ceRNA network brings to light an unknown lncRNA regulatory network in colon cancer. Furthermore, analysis of the ceRNA network identified several lncRNAs that are possibly involved in the regulation mechanisms, progression, and prognosis of colon cancer. Future functional investigation of these lncRNAs is essential to confirm the association with colon cancer and to explore novel potential targets for therapy.

## Figures and Tables

**Table 1:** The competing genes of lncRNAs in the ceRNA network.

lncRNA	Degree	Gene 1	Gene 2	Gene 3	Gene 4	Gene 5	Gene 6	Gene 7	Gene 8	Gene 9	Gene 10	Gene 11	Gene 12	Gene 13	Gene 14	Gene 15	Gene 16	Gene 17	Gene 18
RP11-284N8.3	18	PIK3CG	SEMA6A	IGF1	THRB	DPYD	IL6R	LPAR1	CXCL12	FAS	MT2A	PPARGC1A	CROT	CPEB3	FGF9	ESR1	BCL2L11	KAT2B	MXD1
RP11-399O19.9	17	FAS	CYP19A1	IL10	CASP7	BMP2	CDKN1A	BCL2L11	TNFSF12	NPAS3	HSPB2	TP53INP1	BCL2	EPAS1	KAT2B	PHLPP2	PRKG1	KIT	
LINC00641	13	LPAR1	CXCL12	ATAT1	FAS	PPARGC1A	IL6R	SOX5	FGF7	BCL2	RECK	AKT3	BRCA1	FGFR1					
MAGI2-AS3	12	CXCL12	LPAR1	IL6R	FAS	MT2A	ESR1	CSF1	MEOX2	ZEB1	PDGFRA	TP53INP1	BCL2						
RP11-2C24.4	10	CCNE1	AXIN2	VEGFA	CCND1	CHEK1	BIRC5	CDK4	CDC25A	BRCA1	HMGA1								
STAG3L5P-PVRIG2P-PILRB	10	BRCA1	HMGA1	VEGFA	AXIN2	CCNE1	CCND1	CHEK1	BIRC5	CDC25A	SALL4								
PWAR6	9	CPEB3	ESR1	MEIS1	ZEB2	ZEB1	MITF	CPEB1	NPAS3	TP53INP1									
RP11-305E6.4	9	EZH2	RCBTB1	CHEK1	CKS2	DNMT3B	HMGA1	MYC	CCNE1	CDC6									
RP11-458F8.4	8	CDC25A	CCND1	HMGA1	TNFRSF10B	E2F1	EZH2	MYC	AURKB										
RP13-942N8.1	8	UBE2C	RBL1	MYC	LIMK1	PTPRO	CCND1	VEGFA	E2F1										
GAS5	7	EZH2	CKS2	HMGA1	RCBTB1	CHEK1	IARS	TRIB3											
TRG-AS1	7	HMOX1	NRP2	DYRK2	CCR7	RAVER2	VDR	CDKN1A											
ATP2B1-AS1	6	ZEB2	PTGER4	MEIS1	ZEB1	CPEB1	MITF												
LINC-PINT	6	E2F5	PROX1	RCBTB1	CHEK1	EZH2	ESR1												
AC010226.4	5	PIK3CG	THRB	SEMA6A	IGF1	DPYD													
RP11-545I5.3	5	CXCL12	LPAR1	PPARGC1A	FAS	IL6R													
RP11-834C11.4	5	COL1A2	DYRK2	ITGB3	CCR7	VDR													
LINC01197	4	NOTCH3	SLC8A1	MYOCD	HAND2														
RP11-120E11.2	3	CPEB3	FGF9	ESR1															
KCNQ1OT1	2	ESR1	CYP19A1																
MALAT1	2	ESR1	NPAS3																
RP11-588 K22.2	2	SNCG	CAV1																
RP11-6O2.3	2	JPH2	MXI1																
RP11-798M19.6	2	SNCG	CAV1																
RP1-239B22.5	2	CDC25A	CDC25C																
UCA1	2	HSPD1	TKT																
ALMS1-IT1	1	E2F5																	
LINC00294	1	CDKN1A																	
MIR17HG	1	CCNA2																	
PVT1	1	GPX2																	
RP11-29G8.3	1	KIF20B																	

**Table 2:** Differential expression analysis of lncRNAs in tumor vs. normal tissues and in early-stage vs. late-stage tumors in the network.

lncRNA		Tumor vs. normal			Stages III and IV vs. stages I and II		
lncRNA_ID	lncRNA	logFC	*P* value	FDR	logFC	*P* value	FDR
ENSG00000281103	TRG-AS1	-1.423908012	2.35883*E* − 17	2.06529*E* − 16	-0.493426442	3.09472*E* − 05	0.001872111
ENSG00000261438	RP11-399O19.9	-1.076885782	1.78653*E* − 12	1.03514*E* − 11	-0.422363333	5.85155*E* − 05	0.002745267
ENSG00000214049	UCA1	3.895863041	5.24328*E* − 18	4.83645*E* − 17	0.698484894	0.000322105	0.009535683
ENSG00000273142	RP11-458F8.4	1.199175473	2.69225*E* − 15	2.0399*E* − 14	0.218373189	0.010661844	0.090116662
ENSG00000260196	RP1-239B22.5	1.162274768	1.16143*E* − 15	9.04862*E* − 15	0.187325891	0.02032783	0.132090353
ENSG00000230002	ALMS1-IT1	1.425424943	2.04239*E* − 11	1.05288*E* − 10	0.257786466	0.020982907	0.135386216

**Table 3:** GO function for lncRNA RP11-284N8.3.

GO ID	GO function	*P* value	FDR
60526	Prostate glandular acinus morphogenesis	1.99*E* − 05	6.78*E* − 03
60527	Prostate epithelial cord arborization involved in prostate glandular acinus morphogenesis	1.99*E* − 05	6.78*E* − 03
23033	Signaling pathway	3.28*E* − 05	6.78*E* − 03
48608	Reproductive structure development	3.44*E* − 05	6.78*E* − 03
8584	Male gonad development	4.81*E* − 05	6.78*E* − 03
60736	Prostate gland growth	5.95*E* − 05	6.78*E* − 03
35468	Positive regulation of signaling pathway	6.12*E* − 05	6.78*E* − 03
60442	Branching involved in prostate gland morphogenesis	7.27*E* − 05	6.78*E* − 03
60525	Prostate glandular acinus development	7.27*E* − 05	6.78*E* − 03
10647	Positive regulation of cell communication	9.09*E* − 05	7.09*E* − 03
46546	Development of primary male sexual characteristics	9.29*E* − 05	7.09*E* − 03
10740	Positive regulation of intracellular protein kinase cascade	1.15*E* − 04	7.80*E* − 03
46661	Male sex differentiation	1.21*E* − 04	7.80*E* − 03
30879	Mammary gland development	1.39*E* − 04	8.34*E* − 03
48522	Positive regulation of cellular process	1.71*E* − 04	8.99*E* − 03
23052	Signaling	1.78*E* − 04	8.99*E* − 03
42592	Homeostatic process	1.82*E* − 04	8.99*E* − 03
10907	Positive regulation of glucose metabolism	2.01*E* − 04	9.28*E* − 03
9967	Positive regulation of signal transduction	2.65*E* − 04	9.28*E* − 03
10676	Positive regulation of cellular carbohydrate metabolism	2.75*E* − 04	9.28*E* − 03
45913	Positive regulation of carbohydrate metabolism	2.75*E* − 04	9.28*E* − 03
23056	Positive regulation of signaling process	2.83*E* − 04	9.28*E* − 03
48878	Chemical homeostasis	2.90*E* − 04	9.28*E* − 03
8633	Activation of proapoptotic gene products	3.03*E* − 04	9.28*E* − 03
42981	Regulation of apoptosis	3.08*E* − 04	9.28*E* − 03
43067	Regulation of programmed cell death	3.24*E* − 04	9.28*E* − 03
2690	Positive regulation of leukocyte chemotaxis	3.31*E* − 04	9.28*E* − 03
42531	Positive regulation of tyrosine phosphorylation of STAT protein	3.31*E* − 04	9.28*E* − 03
10941	Regulation of cell death	3.38*E* − 04	9.28*E* − 03
3006	Reproductive developmental process	3.41*E* − 04	9.28*E* − 03
35295	Tube development	3.50*E* − 04	9.28*E* − 03
60740	Prostate gland epithelium morphogenesis	3.61*E* − 04	9.28*E* − 03
48518	Positive regulation of biological processes	3.65*E* − 04	9.28*E* − 03
32787	Monocarboxylic acid metabolism	3.82*E* − 04	9.45*E* − 03
60512	Prostate gland morphogenesis	4.25*E* − 04	9.86*E* − 03
8406	Gonad development	4.34*E* − 04	9.86*E* − 03
30335	Positive regulation of cell migration	4.34*E* − 04	9.86*E* − 03

## Data Availability

The data used to support the findings of this study are available from the corresponding author upon request.

## Supplementary Materials

Supplementary 1 Table S1: target sequences for siRNAs for the top 5 lncRNAs in the colon cancer-specific ceRNA network. Table S2: primer sequence for the QRT-PCR analysis of the top five lncRNAs in the colon cancer-specific ceRNA network. Table S3: specific gene primers of the target genes of lncRNAs. Table S4: specific primers of the target miRNAs of lncRNAs. Table S5: network characteristics of a colon cancer-specific ceRNA network. Figure S1: a flowchart of ceRNA network construction and analysis in our study. Figure S2: verification of the expression of the target miRNAs in the ceRNA network. Figure S3: functional enrichment of lncRNA RP11-399O19.9 in the ceRNA network.

Supplementary 2 Doc S1: characteristics of the patients in the TCGA COAD database.

Supplementary 3 Doc S2: ceRNA crosstalk identified in the COAD database.

Supplementary 4 Doc S3: GO enrichment analysis of the ceRNA network.

Supplementary 5 Doc S4: KEGG pathway analysis of the ceRNA network.

## References

[1] Center M. M., Jemal A., Smith R. A., Ward E. (2009). Worldwide variations in colorectal cancer. *CA: A Cancer Journal for Clinicians*.

[2] Hanahan D., Weinberg R. A. (2011). Hallmarks of cancer: the next generation. *Cell*.

[3] Koirala P., Huang J., Ho T. T., Wu F., Ding X., Mo Y. Y. (2017). LncRNA AK023948 is a positive regulator of AKT. *Nature Communications*.

[4] Rasmussen M. H., Lyskjær I., Jersie-Christensen R. R. (2016). *miR-625-3p* regulates oxaliplatin resistance by targeting MAP2K6-p38 signalling in human colorectal adenocarcinoma cells. *Nature Communications*.

[5] Chakravarty D., Sboner A., Nair S. S. (2014). The oestrogen receptor alpha-regulated lncRNA NEAT1 is a critical modulator of prostate cancer. *Nature Communications*.

[6] Patel N., Morris A., Patel R., Carter G., Cooper D., Murr M. (2015). PKC*δ*VIII and Bcl2 increase ovarian cancer survival via lncRNA NEAT1 secreted by obese adipose derived stem cells. *The FASEB Journal*.

[7] Yu H., Xu Q., Liu F., Ye X., Wang J., Meng X. (2015). Identification and validation of long noncoding RNA biomarkers in human non-small-cell lung carcinomas. *Journal of Thoracic Oncology*.

[8] Zeng C., Xu Y., Xu L. (2014). Inhibition of long non-coding RNA NEAT1 impairs myeloid differentiation in acute promyelocytic leukemia cells. *BMC Cancer*.

[9] Salmena L., Poliseno L., Tay Y., Kats L., Pandolfi P. P. (2011). A *ceRNA* hypothesis: the Rosetta Stone of a hidden RNA language?. *Cell*.

[10] Cesana M., Cacchiarelli D., Legnini I. (2011). A long noncoding RNA controls muscle differentiation by functioning as a competing endogenous RNA. *Cell*.

[11] Karreth F. A., Pandolfi P. P. (2013). ceRNA cross-talk in cancer: when ce-bling rivalries go awry. *Cancer Discovery*.

[12] Taulli R., Loretelli C., Pandolfi P. P. (2013). From pseudo-ceRNAs to circ-ceRNAs: a tale of cross-talk and competition. *Nature Structural & Molecular Biology*.

[13] Poliseno L., Salmena L., Zhang J., Carver B., Haveman W. J., Pandolfi P. P. (2010). A coding-independent function of gene and pseudogene mRNAs regulates tumour biology. *Nature*.

[14] Zhang Y., Li Y., Wang Q. (2017). Identification of an lncRNA-miRNA-mRNA interaction mechanism in breast cancer based on bioinformatic analysis. *Molecular Medicine Reports*.

[15] Xia T., Liao Q., Jiang X. (2014). Long noncoding RNA associated-competing endogenous RNAs in gastric cancer. *Scientific Reports*.

[16] Kun S., Duan Q., Liu G., Lu J. M. (2017). Prognostic value of DNA repair genes based on stratification of glioblastomas. *Oncotarget*.

[17] Wang H., Fu Z., Dai C. (2016). LncRNAs expression profiling in normal ovary, benign ovarian cyst and malignant epithelial ovarian cancer. *Scientific Reports*.

[18] Naderi E., Mostafaei M., Pourshams A., Mohamadkhani A. (2014). Network of microRNAs-mRNAs interactions in pancreatic cancer. *BioMed Research International*.

[19] Yang S., Ning Q., Zhang G., Sun H., Wang Z., Li Y. (2016). Construction of differential mRNA-lncRNA crosstalk networks based on ceRNA hypothesis uncover key roles of lncRNAs implicated in esophageal squamous cell carcinoma. *Oncotarget*.

[20] Tomczak K., Czerwinska P., Wiznerowicz M. (2015). The Cancer Genome Atlas (TCGA): an immeasurable source of knowledge. *Współczesna Onkologia*.

[21] The Cancer Genome Atlas Research Network, Weinstein J. N., Collisson E. A. (2013). The Cancer Genome Atlas Pan-Cancer analysis project. *Nature Genetics*.

[22] Chou C. H., Chang N. W., Shrestha S. (2016). miRTarBase 2016: updates to the experimentally validated miRNA-target interactions database. *Nucleic Acids Research*.

[23] Paraskevopoulou M. D., Vlachos I. S., Karagkouni D. (2016). DIANA-LncBase v2: indexing microRNA targets on non-coding transcripts. *Nucleic Acids Research*.

[24] Hazra D. K., Sriramkumar L., Martin J. (2013). BINGO: a code for the efficient computation of the scalar bi-spectrum. *Journal of Cosmology and Astroparticle Physics*.

[25] Yu G., Wang L. G., Han Y., He Q. Y. (2012). clusterProfiler: an R package for comparing biological themes among gene clusters. *OMICS: A Journal of Integrative Biology*.

[26] Paci P., Colombo T., Farina L. (2014). Computational analysis identifies a sponge interaction network between long non-coding RNAs and messenger RNAs in human breast cancer. *BMC Systems Biology*.

[27] Tay Y., Rinn J., Pandolfi P. P. (2014). The multilayered complexity of ceRNA crosstalk and competition. *Nature*.

[28] Xu J., Li Y., Lu J. (2015). The mRNA related ceRNA–ceRNA landscape and significance across 20 major cancer types. *Nucleic Acids Research*.

[29] Liu J., Wang W., Zhou X. (2014). Construction and investigation of breast-cancer-specific ceRNA network based on the mRNA and miRNA expression data. *IET Systems Biology*.

[30] Sumazin P., Yang X., Chiu H. S. (2011). An extensive microRNA-mediated network of RNA-RNA interactions regulates established oncogenic pathways in glioblastoma. *Cell*.

[31] Yang W., Ning N., Jin X. (2017). The lncRNA H19 promotes cell proliferation by competitively binding to miR-200a and derepressing *β*-catenin expression in colorectal cancer. *BioMed Research International*.

[32] Han Y., Yang Y. N., Yuan H. H. (2014). UCA1, a long non-coding RNA up-regulated in colorectal cancer influences cell proliferation, apoptosis and cell cycle distribution. *Pathology*.

[33] Takahashi Y., Sawada G., Kurashige J. (2014). Amplification of *PVT-1* is involved in poor prognosis via apoptosis inhibition in colorectal cancers. *British Journal of Cancer*.

[34] Yin D., He X., Zhang E., Kong R., De W., Zhang Z. (2014). Long noncoding RNA GAS5 affects cell proliferation and predicts a poor prognosis in patients with colorectal cancer. *Medical Oncology*.

[35] Zheng H. T., Shi D. B., Wang Y. W. (2014). High expression of lncRNA MALAT1 suggests a biomarker of poor prognosis in colorectal cancer. *International Journal of Clinical and Experimental Pathology*.

[36] Zheng Y., Song D., Xiao K. (2016). LncRNA GAS5 contributes to lymphatic metastasis in colorectal cancer. *Oncotarget*.

[37] Wu Q., Meng W. Y., Jie Y., Zhao H. (2018). LncRNA MALAT1 induces colon cancer development by regulating miR-129-5p/HMGB1 axis. *Journal of Cellular Physiology*.

[38] Yu X., Zhao J., He Y. (2018). Long non-coding RNA PVT1 functions as an oncogene in human colon cancer through miR-30d-5p/RUNX2 axis. *Journal of Balkan Union of Oncology*.

[39] Ping G., Xiong W., Zhang L., Li Y., Zhang Y., Zhao Y. (2018). Silencing long noncoding RNA PVT1 inhibits tumorigenesis and cisplatin resistance of colorectal cancer. *American Journal of Translational Research*.

[40] Liao Q., Liu C., Yuan X. (2011). Large-scale prediction of long non-coding RNA functions in a coding-non-coding gene co-expression network. *Nucleic Acids Research*.

[41] Ma H., Hao Y., Dong X. (2012). Molecular mechanisms and function prediction of long noncoding RNA. *The Scientific World Journal*.

[42] Guo Q., Cheng Y., Liang T. (2015). Comprehensive analysis of lncRNA-mRNA co-expression patterns identifies immune-associated lncRNA biomarkers in ovarian cancer malignant progression. *Scientific Reports*.

[43] Qiu J. J., Lin Y. Y., Ye L. C. (2014). Overexpression of long non-coding RNA HOTAIR predicts poor patient prognosis and promotes tumor metastasis in epithelial ovarian cancer. *Gynecologic Oncology*.

[44] Yang F., Zhang L., Huo X. S. (2011). Long noncoding RNA high expression in hepatocellular carcinoma facilitates tumor growth through enhancer of zeste homolog 2 in humans. *Hepatology*.

